# Social networks and their influences on nutrient intake, nutritional status and physical function in community-dwelling ethnically diverse older adults: a mixed-methods longitudinal study

**DOI:** 10.1186/s12889-020-09153-y

**Published:** 2020-06-26

**Authors:** Evans A. Asamane, Carolyn A. Greig, Janice L. Thompson

**Affiliations:** 1 School of Sports, Exercise and Rehabilitation Sciences, College of Life and Environmental Sciences, University of Birmingham, Birmingham, UK; 2 School of Primary, Community and Social Care, Keele University, Keele, UK; 3 NIHR Birmingham Biomedical Research Centre, University Hospitals Birmingham NHS Foundation Trust and University of Birmingham, Birmingham, UK

**Keywords:** Social networks, Ethnic minority, Diversity, Super-diverse, Physical function, Nutrients, Nutritional status, Qualitative

## Abstract

**Background:**

The United Kingdom population is ageing and becoming increasingly diverse; thus, it is vital to develop and implement interventions supporting this population shift. Social networks (SN) significantly impact health outcomes in later life, however relatively little is known about SN of community-dwelling ethnically diverse older adults. This study aimed to: 1) profile SN and changes in SN in this population over 8 months; 2) examine associations between SN, dietary intake, nutritional status, and physical function.

**Methods:**

SN were assessed using the Wenger Practitioner Assessment of Network Type. Energy and nutrient intakes were measured using multiple-pass 24-h recalls. The Mini Nutritional Assessment-Short Form (MNA-SF) assessed nutritional status. Physical function was measured using the Short Physical Performance Battery (SPPB) and handgrip strength. Data were collected at baseline and 8-months. Correlation and regression analyses examined relationships between SN, physical function, nutrient intake and nutritional status. Semi-structured interviews were conducted at baseline (*n* = 92) and follow-up (*n* = 81) to identify potential influences of SN. Interviews were transcribed verbatim and analysed using directed content analysis.

**Results:**

Quantitative data were obtained from 100 participants at baseline and 81 at follow-up. Mean (SD) age was 70.8 (8.1) years (59% male), comprising African/Caribbean (60%), South Asian (34%), and other ethnicities (6%). Five SN typologies were identified under two broad areas: integrated-SN consisting of locally integrated (44%) and wider community (8%); and non-integrated-SN consisting of family dependent (25%), local self-contained (17%), and private restricted (6%). At follow-up, 37% remained in non-integrated networks, 19% transitioned to non-integrated networks, 11% transitioned to, and 33% remained in, integrated networks. Participants within integrated networks at baseline had higher SPPB scores at follow-up. Compared to the private restricted, local self-contained SN significantly predicted zinc, riboflavin and vitamin B6 intakes. Participants remaining in, or transitioning to, non-integrated networks had low MNA-SF scores. Qualitative findings indicate that participants with reductions in SN perceived it as causing poorer physical function and eating behaviours.

**Conclusion:**

In the present study, integrated SN were associated with higher physical function and nutritional status at 8-month’s follow-up. These results can inform the design of interventions to improve social networks, physical function and healthy nutrition within this population.

## Background

Social networks are described as inter-personal ties and relationships shared among a group of inhabitants in an environment [1, 2]. These ties often offer a sense of identity, support, solidarity and feelings of belonging [3, 4]. During later life, social networks are unstable, often with severe disruptive changes such as bereavement, and are more likely to become smaller as compared to middle life [5]. These disruptions, as explained by the convoy model of social networks, are mainly caused by situational and personal characteristics such as retirement, ill-health, loss of a spouse, residential changes and other life events primarily associated with ageing [5, 6]. Evidence suggests that stronger social networks positively influence quality of life, morbidity and mortality in later life [7–10]. A study examining the influence of social networks on health outcomes among 4170 Korean adults aged 65 years and older found that individuals with more extensive social networks had higher levels of life satisfaction and lower depressive symptoms than those with restricted networks [11]. Additionally, an international study of 13,891 older adults in eight developing countries discovered that individuals with integrated social networks were more protected against premature all-cause mortality over three and half years of follow-up as compared to those in restricted social networks [9]. Given this, it is necessary to understand the types of social networks among diverse populations living in diverse communities, and how these social networks influence healthy lifestyle choices over time.

The UK population is ageing and becoming more ethnically diverse [12]. Ethnic minorities account substantially for the recent population growth, and are estimated to constitute over 30% of the population by 2050 [12, 13]. The majority of ethnic minorities live in areas of higher deprivation and experience disproportionate health inequalities, predisposing them to a lower quality of life and poorer health than the White British population [14, 15]. Even though policies and interventions have been implemented to try to address this gap, much still needs to be done to support this rapidly growing portion of the population to age more healthily [16]. A more in-depth enquiry of social networks in this population could contribute to a wider understanding of the relationship between social networks and health, and thus help in developing more culturally sensitive community-based interventions with the potential to improve social networks, and thereby help address the health disparities among this population. An example of such culturally sensitive community-based interventions could include, but are not limited to, health professionals and researchers collaborating with ethnic specific community centres/age well societies and religious organisations to co-create healthy messages and other activities that can be delivered as outreach programmes at these centres. These types of interventions could increase awareness and promote behaviour change, as well as improve attendance at these group sessions. This has the potential of improving social networks and subsequent health and wellbeing among this population.

It is often assumed that ethnic minorities live in multigenerational households that offer continuous informal support for their older members, and hence have stronger social networks than White older adults. However, this is often not the case, as growing evidence suggests that multigenerational households do not translate into a continuous pool of support [17]. Additionally, there is evidence that ethnic minorities do not have extensive social networks or a lower risk of loneliness as previously reported [18–21]. For example, a longitudinal study exploring social networks and engagement between Blacks and Whites aged 65 years and older in the United States (US) found that Blacks had smaller social networks and engagements as compared to Whites over the study period [21]. While recognising these disparities between ethnic minorities and the predominately White population, there is also evidence pointing to differences in social networks among ethnic minorities themselves. For instance, a recent study of social networks of immigrants aged 55 years and older in England and Wales found that Black Caribbean and Chinese older immigrants had a higher proportion of restricted non-kin social networks, at 30 and 22.3%, respectively, as compared to Pakistanis (12.1%), Bangladeshis (6.7%), Indians (16.3%) and Black Africans (16.8%) [22]. These differences within and between ethnic minorities and White populations further cast doubt on the assumptions that the majority of ethnic minorities have extensive social networks. These studies also provide a rationale to explore and compare the social networks of older adults living in a super-diverse city, with that of previous studies of ethnic minorities elsewhere. This could be useful in broadening our understanding of social networks of ethnic minorities living in different environmental contexts, and subsequently aid in the design and implementation of tailored interventions to improve social networks.

There are relatively few studies that have explored the role of social networks on health outcomes of ethnic minorities in later life [22–25]. These studies have highlighted the critical relationship between social networks and health outcomes. For example, in the US, a cross-sectional study involving 700 South Asians aged 44–84 years found that increases in emotional closeness of network members was associated with better self-rated health [23]. Similarly, in the UK, a study involving six ethnic minority groups (aged 55+ years) using quantitative measures within a cross-sectional study design, found that older ethnic migrants with more extensive family-oriented networks reported lower loneliness and higher quality of life than those in restricted networks [22]. Even though these studies have advanced our understanding of the relationship of social networks and some health outcomes within ethnic minorities, they are often cross-sectional in design, thus making it difficult to monitor changes in social networks, and how these changes may subsequently influence health outcomes.

A longitudinal study design enables the disentangling of the temporality of associations and helps to unravel the influence of changes in social networks on health outcomes over time. Furthermore, considering the potential complex mechanisms of how social networks exert influence, the use of only quantitative techniques, as done in previous studies, limits us from understanding why these changes occur or how they might be influencing health. To address these gaps in the literature, the present study uses a longitudinal concurrent mixed method approach aimed to: 1) categorise and assess any changes in the social network profiles of community-dwelling ethnically diverse older adults over 8-months; and 2) examine the extent to which social networks, and any changes in these networks, influence nutrient intake, nutritional status and physical function over 8-months.

## Methods

### Study design and study setting

**Fig. 1:**
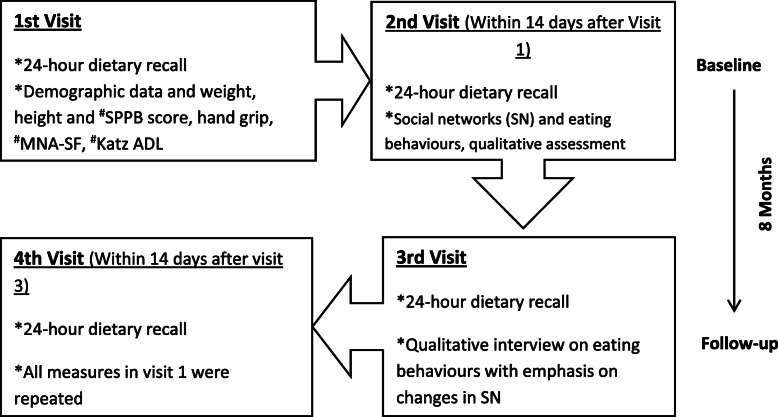
Flow chart of recruitment, data collection and reasons for dropouts

A longitudinal concurrent mixed method approach was used to explore social networks and their associations with nutrient intake, nutritional status and physical function at baseline and 8-month follow-up [26]. Data were collected on four different occasions: two visits at baseline (T1) and two visits at follow-up (T2) (see Fig. 1). The two visits within each time point were carried out across a 14-day period to capture a closer representation of habitual intake of food [27]. At each of these visits, both quantitative and qualitative data were collected. All baseline data were collected from March 2017 to February 2018, and follow-up data collection occurred approximately 8 months later, from November 2017 to February 2019. The study was conducted in Birmingham, UK, which is the second most populated city in the UK. Recent projections predict that by 2021, more than 50% of the population in Birmingham will be comprised of ethnic minorities [28]. Given this, the present study setting was appropriate to answer the research questions in this study.

### Participants and recruitment

**Fig. 2:**
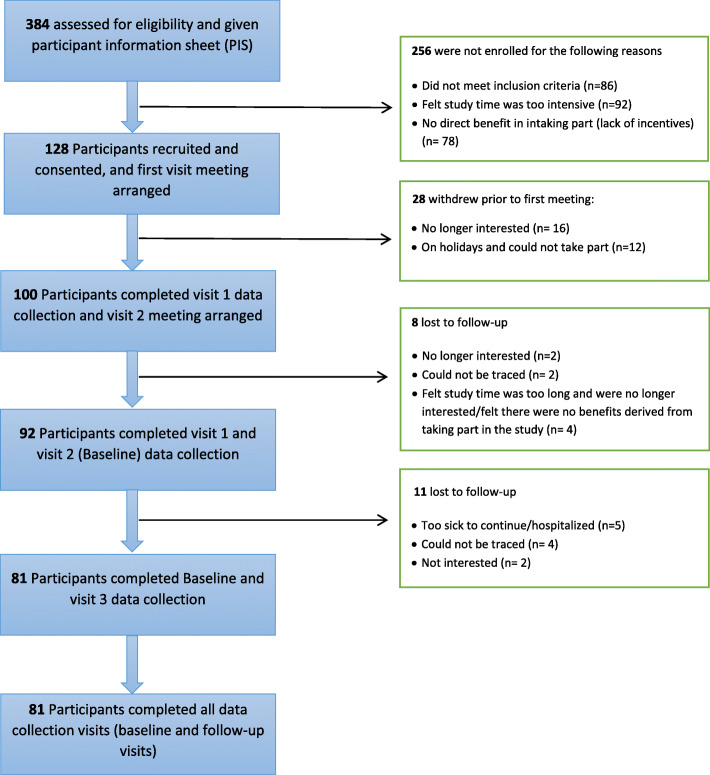
*Flow chart summarising the data collection phases and visits over the 8-month period.*^#^SPPB = Short Performance physical battery; MNA-SF = Mini Nutritional Status-Short Form; Katz-ADL = Katz Activity of Daily Living

Community-dwelling ethnically diverse older adults (≥60 years), living in Birmingham, and self-identifying as African, Indian, Pakistani, Bangladeshi or Caribbean, took part in the study. As shown in Fig. 2, a total of 384 potential eligible participants were approached and provided with a participant information sheet (PIS) at age-well societies, community centres, faith centres and other informal social events across Birmingham. Of this number, 100 participants were conveniently and purposively recruited and consented to take part in the study with all baseline data collected. The participation rate was 26%, which is defined in this study as the total number of eligible consented participants in the study at baseline divided by the total number of potential participants that were approached and received a PIS. Recruitment excluded older adults with a diagnosis of dementia or any cognitive disabilities that might affect their participation. Using the Standard Mini Mental State Examination (SMMSE) test, a score of < 19 was considered moderately cognitively impaired and hence not eligible to take part in the study [29, 30]. Furthermore, older adults that were institutionalised or hospitalised were excluded. Maximum variation sampling and chain referrals were employed during the recruitment process to ensure broad representation across older age groups, level of deprivation, ethnicities, sex and religions/faiths groups [31–33]. Several techniques were incorporated to increase recruitment, improve data quality and increase participant retention in the study. The authors established initial contact with community leaders and potential participants prior to the start of the study. In addition, EAA (Evans A. Asamane) attended church services and public events held at faith centres to develop rapport with community leaders and potential participants. This may have enhanced trust between EAA and potential participants, which has been shown to improve the quality of data collection [34]. Community leaders who met the inclusion criteria were also encouraged to take part in the study. By recruiting those serving as role models, more trust and credibility was given to the study [34].

The study received ethical approval from the University of Birmingham Science, Technology, Engineering, and Mathematics (STEM) ethical review committee (ERN_17_1364). All participants provided written informed consent before data were collected.

### Data collection

#### Socio-demographic characteristics

Socio-demographic data of participants including date of birth, sex, marital status, educational status, faith, ethnicity, and years residing in the UK were collected using a bespoke questionnaire, at baseline and at follow-up. Additionally, using this bespoke questionnaire, self-reported health, number of diseases and medication use by participants were gathered. This questionnaire also captured participants’ postcodes which were used to generate the Index of Multiple Deprivation (IMD), an indicator of the level of deprivation across England [35].

### Social networks

Among the different social network typologies, two social network generation tools were considered appropriate for this study: the Burholt et al. social network typology for people living in multigenerational households and the Wenger Practitioner Assessment of Network Type (PANT) [36–38]. Although somewhat similar, these network typologies differ in the number of network groups, the composition of typology and the ease of applicability [36]. For instance, the Burholt typology uses nine ‘network generator’ questions to derive four network groups and has been tested and validated to more accurately identify restricted social networks among people living in multigenerational households than the Wenger PANT [36]. The Wenger PANT includes five network groups, is easy to administer, and has been validated and used in many populations in different countries, including ethnic minority populations in the UK [39–42]. Although data were collected using these two network typologies in the present study, the Wenger PANT was used to categorise older adults’ social networks because of the low proportion (5%) of multigenerational households in the study sample and its ease of administration.

The Wenger PANT is composed of eight distinct questions based on the availability of close local kin (3 questions), the closeness of family, friends and neighbours (3 questions), and level of interaction with community, voluntary and religious groups (2 questions) [37, 38]. This scoring tool generates five social network types that describe the size, composition and function of the social network. These five social networks include: locally integrated; wider community focussed; family dependent; local self-contained; and the private restricted networks. These are summarised below under two broad categories, integrated and non-integrated social networks:

#### Integrated social networks

1. The locally integrated social network is considered more robust and integrated, providing an optimal level of support as compared to the other network types. It is often larger than average (network size more than 8 members) and involves active involvement with family, friends, neighbours and the wider community. This is the most common network found in the UK [40, 43].
2. The wider community focussed network is also large (more than 8 members within the network). It is characterised by high community involvement and no local kin [43]. Family members are always a distance from the individual but might visit occasionally. There is a high dependence on friends and neighbours, and with low levels of isolation [43].

#### Non-integrated social networks

3. The family dependent network is typically characterised by a small network size (1–4 members). It has high family involvement and low involvement with the community. Individuals are usually living with an adult child or relative and are often widowed. The family dependent social network has a high dependence on family members [40, 43].
4. The local self-contained network is characterised by low community involvement, and family members might be present locally with low involvement. Individuals within this network are generally reliant on neighbours for support. This network size tends to be smaller than average (5–6 members), and presents high levels of isolation [40, 43].
5. The private restricted social network is less common in the UK as compared to the other social networks; it is characterised by very low involvement with family, friends, and the wider community [43]. There are usually no local kin, and individuals within this network are at high risk of isolation, loneliness, depression and low quality of life in general [43].

### Energy and nutrient intake

Repeated multiple pass 24-h dietary recalls coupled with qualitative interviews were used to assess energy and nutrient intakes on four different occasions (2 non-consecutive days each at baseline and follow-up), including at least one weekend day following standard procedures [44]. EAA, a trained nutritionist with relevant expertise in conducting dietary assessments, conducted all assessments with oversight from JLT (Janice Lee Thompson), a Professor of Public Health Nutrition and Exercise, who has extensive experience in dietary assessment among different populations. A culturally sensitive full-colour food portion booklet containing over 1700 ethnically diverse foods eaten in the UK was used to aid in portion size estimations [45]. The use of dietary supplements was captured during the multiple pass 24-h recalls. 24-h recall records were discarded when participants indicated that the previous day was not habitual, or they had fasted (*N* = 17), and an additional data collection session was completed to obtain the record. Before data entry, recalls were cross-checked for accuracy using the digital audio-recorded interviews before entering data into the Dietplan 7.0 dietary software analysis package (Forestfield Software Ltd. Horsham, UK) for processing. West African food composition tables, food manufacturing websites, and recipe books for ethnic minority groups were employed when a food item could not be identified in the Dietplan 7.0 database [46].

### Nutritional status

Participants’ nutritional status were assessed using the Mini Nutritional Assessment-Short Form (MNA-SF). This is a simple to use, sensitive, reliable and valid tool for rapid assessment of the nutritional status of older adults [47]. Unlike the full MNA, the short form is faster to complete and equally reliable when compared to the full MNA [48, 49]. Considering its ease of administration, this was the preferred tool to assess nutritional status in the present study as it reduces participant burden. Furthermore, most of the information on the full MNA (such as dietary information) was collected using the multiple pass 24-h recall, further justifying the use of the short version. The MNA-SF is composed of six major questions and was administered at visits 1 and 3 (see Fig. 1). The MNA-SF has a scoring of 0–14 points, with as score of 0–7 classified as the malnourished group, 8–11 classified as at-risk-of malnutrition group, and 12–14 classified as normal group [49].

### Anthropometrics and physical function

Body weight was measured using a Seca 899 digital scale to the nearest 0.1 kg, with participants wearing light clothing and no shoes. Height was measured using a Seca 213 portable stadiometer to the nearest 0.1 cm, with participants wearing no shoes/minimal headwear. Body Mass Index (BMI) was calculated using the standard equation, weight (kg)/height (m)^2^. Waist circumference was measured using a flexible retractable non-elastic tape and recorded to the nearest 0.01 cm.

The Short Physical Performance Battery (SPPB) was used to measure participants’ lower body extremity physical function [50–52]. These measures include gait speed, balance and time taken to perform five repeated chair rises [52]. Handgrip strength was measured using a Jamar hand dynamometer in the standing position [53]. The standing position was chosen as it has been shown to produce maximal grip strength as compared to other body positions [54]. During all measurements, the Jamar handgrip dynamometer was adjusted to the second handle as this has also been shown to produce consistent and reliable maximal handgrip strength results [55]. Each measurement was completed three times and the highest recording used for analysis.

### Quantitative analysis

SPSS version 25 (IBM Corp, Armonk, NY, 2017) was used to carry out all statistical analyses, with significance set at a *p*-value set of 0.05. Bivariate analyses were conducted to ascertain the difference between the baseline sample and the sample lost to follow-up (*n* = 18). Descriptive analyses were conducted to explore the frequencies and percentages of variables of interest by social networks. Paired t-tests and chi-square tests were used to test for differences between continuous variables and categorical variables, respectively. In instances where normality was violated, the Wilcoxon signed-rank test was used instead of the paired t-test. One-way ANOVA, with the addition of the Bonferroni multiple comparisons test, was used to test the differences in continuous variables between social networks. Hierarchical multiple linear regressions were used to assess the association of SN with nutritional status, nutrients intake and physical function (SPPB and handgrip strength) at baseline. At follow-up, the influence of baseline SN on physical function and nutritional status at follow-up was also ascertained using multiple linear regressions.

Social network typologies were first treated as categorical variables, where dummy variables were created for locally integrated, wider community, family dependent, local self-contained and private restricted networks. The private restricted network was used as the reference category for cross-sectional associations, while the remainder of the four dummy social networks served as predictor variables in unadjusted and adjusted regression models to predict: a) baseline SPPB scores (physical function); b) baseline nutritional status; and c) baseline nutrient intake (including dietary supplements).

Considering previous literature [37, 56] and as described above, the locally integrated and wider community social networks formed the integrated social network type, while family dependent, local self-contained and private restricted social networks formed the non-integrated social network type. Change in social networks was computed as maintenance or change of social network group membership at follow-up in reference to the non-integrated social network group. Four groups were formed: 1) those that remained in the non-integrated social network group; 2) those that changed to the non-integrated social network group; 3) those that changed to the integrated social network group; and 4) those that remained in the integrated social network group. This combination was necessary to run the longitudinal regression given that the five social network memberships reduced due to loss to follow-up. So, for longitudinal analyses, the baseline social network groups (the integrated and non-integrated groups) served as independent predictors in multinomial regression analyses to predict the changes in physical function and nutritional status at follow-up while controlling for age, sex, IMD, self-rated health, number of diseases and educational attainment.

### Qualitative interviews and analysis

A semi-structured interview schedule was used to guide in-depth qualitative interviews at baseline and follow-up, using open-ended questions and following up with relevant probes. At baseline, the interviews were focused on the perceived impact of social networks on eating behaviours and physical function. At the 8-month follow-up, the interview guide was revised to incorporate areas capturing perceived changes in social networks and their potential influences on eating behaviours and physical function. Trained translators were employed in instances where the participant had limited ability to communicate in English (*n* = 19). All interviews were digitally recorded and were 25–110 min in duration. Interviews were transcribed verbatim, then entered into QSR NVivo 12 Plus software for reorganising and coding [57].

Directed content analysis was used to analyse the qualitative data [58, 59]. The directed content analysis approach includes three main steps: preparation, organisation and reporting phases. The preparation phase involved immersion in the data, where transcripts and field notes were read several times to understand the pattern of the data. During the organisation phase, two categorisation matrices were developed: 1) the influence of social networks and other key factors on eating behaviours; and 2) the influence of social networks and other key factors on physical function. Relevant literature and an ecological model of creating healthy environments were used in the initial formation of these matrices [60–62].

Using an iterative process, the matrices were further restructured inductively based on the findings arising (data-driven). To ensure rigour, trustworthiness and the ease of use of the matrices, two independent researchers pre-tested the matrices using preliminary data [58]. Afterwards, discussions around the difficulty of use of the matrices and interpretations of the categories were resolved by consensus, and the matrices further restructured. Following this step, the remaining transcripts were coded by EAA using the updated categorisation matrices. All findings were systematically presented in the reporting phase.

## Results

At baseline, 100 community-dwelling ethnically diverse older adults took part in the study, with a mean (SD) age of 70.8 (8.1) years (59% males). This sample comprised Africans/Caribbean (60%), South Asians (34%) and other ethnicities (6%). More men were married (47%) as compared to women (19%). Seven percent and 9% of men and women respectively were widowed. At follow-up, data were available for 81% of the sample, with a mean (SD) age of 70.9 (8.1) years (62% males). Figure 2 displays the breakdown of recruitment and drop-outs throughout the phases of data collection.

Five different social networks (SN) were identified at baseline and follow-up visit: locally integrated (44% vs 37%), wider community (8% vs 9.9%), family dependent (25% vs 35.8%), local self-contained (17% vs 9.9%), and private restricted (6% vs 7.4%). Participants belonging to private restricted as compared to locally integrated networks had significantly lower SPPB scores (*p* = 0.014). There was a statistically significant association between living arrangements and social networks (*p* = 0.001). Furthermore, as compared to the private restricted network, locally integrated social networks were associated with better self-rated health (*p* = 0.02) (Table 1). (See Additional file 1 for details of socio-demographic characteristics and other variables by the two broad SN categorisation, integrated and non-integrated).

**Table 1:** Demographic characteristics and other variables by social networks at baseline (*N* = 100)

		Integrated SN (***n*** = 52)		Non-integrated SN (***n*** = 48)			
Variable		Locally integrated (***N*** = 44)	Wider community (***N*** = 8)	Family dependent (***N*** = 25)	Local self-contained (***N*** = 17)	Private restricted (***N*** = 6)	***P***-value^**α**^
**Age Mean (SD)**		70.8 (7.8)	75.3 (7.6)	68.5 (8.2)	70.6 (7.2)	74.3 (11.8)	0.230
**Sex N (%)**	Male	28 (63.6)	3 (37.5)	19 (76.0)	6 (35.3)	4 (66.7)	0.045
	Female	16 (36.4)	5 (62.5)	6 (24.0)	11 (64.7)	2 (33.3)	
**Marital Status**^**¥**^	Married	34 (77.3)	4 (50.0)	20 (80.0)	6 (35.3)	2 (33.3)	0.004
	Not married	10 (22.7)	4 (50.0)	5 (20.0)	11 (64.7)	4 (66.7)	
**Ethnicity N (%)**	Caribbean	16 (36.4)	4 (50.0)	6 (24.0)	12 (70.6)	3 (50.0)	0.434
	Pakistani	12 (27.2)	2 (25.0)	6 (24.0)	3 (17.6)	0 (0.0)	
	African	7 (16.0)	1 (12.5)	7 (28.0)	1 (5.9)	3 (50.0)	
	Indian	3 (6.8)	1 (12.5)	2 (8.0)	1 (5.9)	0 (0.0)	
	Bangladeshi	2 (4.5)	0 (0.0)	2 (8.0)	0 (0.0)	0 (0.0)	
	Others^**β**^	4 (9.1)	0 (0.0)	2 (8.0)	0 (0.0)	0 (0.0)	
**Faith/Religion N (%)**	Christian	22 (50.0)	5 (62.5)	13 (52.0)	11 (64.7)	5 (83.3)	0.759
	Muslim	18 (40.9)	2 (25.0)	9 (35.0)	4 (23.5)	1 (16.7)	
	Sikh	3 (6.8)	1 (12.5)	2 (8.0)	1 (5.9)	0 (0.0)	
	Hindu	1 (2.3)	0 (0.0)	1 (4.0)	0 (0.0)	0 (0.0)	
	No religion	0 (0.0)	0 (0.0)	0 (0.0)	1 (5.9)	0 (0.0)	
**Self-R health N (%)**	Excellent/good	35 (79.1)	4 (50.0)	16 (64.0)	7 (44.4)	1 (16.7)	0.005
	Fair/poor	9 (20.9)	4 (50.0)	9 (36.0)	10 (55.6)	5 (83.3)	
**IMD Quartiles N (%)**	1 (Most deprived)	17 (38.6)	2 (25.0)	5 (20.0)	4 (23.5)	5 (83.3)	0.116
	2	7 (15.9)	1 (12.5)	8 (32.0)	3 (17.6)	0 (0.0)	
	3	9 (20.5)	1 (12.5)	5 (20.0)	6 (35.3)	1 (16.7)	
	4 (least deprived)	11 (25.0)	4 (50.0)	7 (28.0)	4 (23.5)	0 (0.0)	
**SPPB Mean (SD)**		9.9 (2.5)	8.6 (3.2)	9.7 (2.8)	7.8 (3.6)	5.7 (4.6)	0.014
**HGS Mean (SD)**		27.7 (8.9)	24 (5.4)	29.1 (9.9)	22.8 (9.4)	26.3 (14.3)	0.231
**MNA-SF Mean (SD)**		12.4 (1.7)	12.8 (1.1)	12.7 (1.7)	12.2 (1.8)	11.5 (3.3)	0.296
BMI categories* N (%)	Underweight	0 (0.0)	0 (0.0)	0 (0.0)	0 (0.0)	0 (0.0)	0.852
	Normal	2 (4.5)	1 (12.5)	2 (8.0)	2 (11.8)	0 (0.0)	
	overweight	13 (29.5)	3 (37.5)	9 (36.0)	4 (23.5)	2 (33.3)	
	Obese	29 (65.9)	4 (50.0)	14 (56.0)	11 (64.7)	4 (66.7)	
**Number of Disease Mean (SD)**		1.8 (1.0)	2.3 (1.5)	2.1 (1.4)	2.5 (1.7)	2.7 (2.4)	0.334
**Diseases n (%)**	Type 2 Diabetes	18 (40.9)	4 (50.0)	15 (60.0)	10 (58.8)	4 (66.7)	0.466
	CVD	10 (22.7)	1 (12.5)	3 (12.0)	4 (23.5)	2 (33.3)	0.688
	Osteoarthritis	10 (22.7)	1 (12.5)	6 (24.0)	6 (35.3)	1 (16.7)	0.742
	High BP	21 (48.8)	5 (62.5)	13 (52.0)	11 (64.7)	3 (60.0)	0.813
	High Cholesterol	5 (11.4)	1 (12.5)	5 (20.0)	1 (5.9)	1 (15.7)	0.735
	Cancer^μ^	4 (9.1)	1 (12.5)	2 (8.0)	0 (0.00)	2 (33.3)	0.191
	Others^∞^	5 (11.4)	1 (12.5)	4 (16.0)	2 (11.8)	1 (16.7)	0.987
**Length of stay in UK**		47.7 (11.9)	50.6 (6.2)	34.7 (20.1)	45.7 (11.3)	20.5 (10.6)	0.001
**Living alone %**		11.6	50.0	8.0	55.6	66.7	0.001
**Supplement use**	Yes	16 (37.2)	3 (37.5)	8 (32.0)	11 (61.1)	1 (16.7)	0.236
	No	27 (62.8)	5 (62.5)	17 (68.0)	7 (38.9)	5 (83.3)	

*SD* Standard deviation; *IMD* Index of Multiple Deprivation; *MNA-SF* Mini Nutritional Assessment-Short Form; *SPPB* Short Physical Performance Battery; *HGS* Handgrip strength; Self-rated health was coded as excellent = 1, good = 2, fair = 3 and poor = 4. These further categorised as 1 = Excellent/Good and 2 = Fair/Poor; BMI = Body Mass Index *WHO guidance on BMI thresholds for Asian populations (World Health Organization, 2004) was used to categorise BMI of South Asian participants, and the standard BMI categories were used for Caribbean and African participants. Note age is in years*.*^**¥**^ This includes all those that are single, separated, divorced and widowed. ^μ^ types of cancer: prostate cancer (70%) and bone cancer (30%); ^∞^Others refers to diseases such kidney diseases, acid reflux, ear and eye problems, and osteoporosis; ^α^ Significant differences between social networks. ^**β ‘**^Others’ referring to mixed ethnicities e.g. African Asians

### Profile of social networks and their changes over eight months

The total proportion of participants reporting locally integrated and local self-contained networks was reduced by 8.7 and 6.1%, respectively, at follow-up (Table 2). However, participants reporting private restricted, wider community and family dependent social networks increased by 1.2, 3.7 and 9.9%, respectively, at follow-up. The most dominant social network was the locally integrated social network, and the least common was the private restricted social network at both time points. Comparing the two time points, locally integrated and family dependent networks were more stable with 62.2 and 66.7%, respectively, at follow-up. At follow-up, 2.7% of those in the locally integrated social network at baseline declined to the local self-contained network, with 27% in the locally integrated social network declining to the family-dependent network. The most unstable network group was the local self-contained, with only 23.1% retaining this category at follow-up. Additionally, 30% of those in the local self-contained network at baseline changed to the private restricted social network at follow-up.

**Table 2:** Profile of social networks and transition over time (N = 81)

		Social networks, follow-up					
Social networks, baseline N (%)		Locally integrated	Wider community	Family dependent	Local self-contained	Private restricted	Total
	Locally integrated	23 (62.2)	3 (8.1)	10 (27.0)	1 (2.7)	0 (0.0)	37 (45.7)
	Wider community	1 (20.0)	2 (40.0)	1 (20.0)	1 (20.0)	0 (0.0)	5 (6.2)
	Family dependent	5 (23.8)	0 (0.0)	14 (66.7)	2 (9.5)	0 (0.0)	21 (25.9)
	Local self-contained	1 (7.7)	2 (15.4)	3 (23.1)	3 (23.1)	4 (30.8)	13 (16.0)
	Private restricted	0 (0.0)	1 (20.0)	1 (20.0)	1 (20.0)	2 (40.0)	5 (6.2)
Total		30 (37.0)	8 (9.9)	29 (35.8)	8 (9.9)	6 (7.4)	81 (100.0)

Table 3 shows the differences in demographic characteristics and outcome variables between those who remained in the integrated network group, those who changed to the integrated network group, those who remained in the non-integrated network group, and those who changed to the non-integrated network group. The groups were not significantly different with respect to age, sex, marital status, number of diseases, handgrip strength or waist circumference. However, the groups differed in MNA-SF scores, SPPB scores, self-rated health and length of stay in the UK. Those who remained in the non-integrated network group had significantly lower SPPB scores at baseline (*P* = 0.039) and follow-up (*p* = 0.018), as compared with participants who remained in the integrated network group. Similarly, at follow-up, participants who changed to the non-integrated network group had the lowest MNA-SF scores (p = 0.018). Participants who remained in the non-integrated network group had lower self-rated health as compared to those who remained in the integrated network (*P* = 0.05).

**Table 3:** The profile of SN changes among community-dwelling ethnically diverse older adults living in Birmingham, UK (*N* = 81)

Variable		Total at follow-up (*N* = 81)	Remained integrated (*N* = 27)	Changed to integrated (*N* = 9)	Remained non-integrated (*N* = 30)	Changed to non-integrated (*N* = 15)	*P*-value*
Age Mean (SD)		70.9 (8.1)	71.3 (7.7)	72.4 (9.1)	69.8 (8.8)	71.3 (7.5)	0.808
Sex N (%)	Male	50 (61.7)	16 (59.3)	6 (66.7)	19 (63.3)	9 (60.0)	0.975
	Female	31 (38.3)	11 (40.7)	3 (33.3)	11 (36.7)	6 (40.0)	
Marital status	Married	55 (67.9)	23 (85.2)	5 (55.6)	18 (60.0)	9 (60.0)	0.137
	Not married	26 (32.1)	4 (14.8)	4 (44.4)	12 (40.0)	6 (40.0)	
Ethnicity N (%)	Pakistani	6 (22.2)	2 (22.2)	2 (6.7)	5 (33.3)	6 (22.2)	0.483
	Indian	2 (7.4)	0 (0.0)	3 (10.0)	0 (0.0)	2 (7.4)	
	Bangladeshi	0 (0.0)	0 (0.0)	2 (6.7)	0 (0.0)	0 (0.0)	
	Caribbean	14 (51.9)	5 (55.6)	14 (46.7)	5 (33.3)	14 (51.9)	
	African	3 (11.1)	1 (11.1)	8 (26.7)	3 (20.0)	3 (11.1)	
	Others	2 (7.4)	1 (11.1)	1 (3.30)	2 (13.3)	2 (7.4)	
IMD Quartiles N (%)	1 (Most deprived)	10 (37.0)	4 (44.4)	10 (33.3)	5 (33.3)	10 (37.0)	0.924
	2	4 (14.8)	2.0 (22.2)	9 (30.0)	4 (26.7)	4 (14.8)	
	3	4 (14.8)	1 (11.1)	5 (16.7)	1 (6.70)	4 (14.8)	
	4 (least deprived)	9 (33.3)	2 (22.2)	6 (20.0)	5 (33.3)	9 (33.3)	
Self-rated health N (%)	Excellent	10 (12.3)	4 (14.8)	2 (22.2)	3 (10.0)	1 (6.7)	0.05
	Good	42 (51.9)	21 (77.8)	4 (44.8)	11 (36.7)	6 (40.0)	
	Fair	18 (22.2)	2 (7.4)	2 (22.2)	8 (26.7)	6 (40.0)	
	Poor	11 (13.6)	0 (0.0)	1 (11.1)	8 (26.7)	2 (13.3)	
Number of diseases Mean (SD)		2.0 (1.4)	1.7 (1.2)	1.6 (0.7)	2.3 (1.7)	2.1 (1.0)	0.303
MNA-SF Mean (SD)	T1	12.6 (1.6)	12.7 (1.4)	13.0 (0.7)	12.5 (2.0)	12.1 (1.0)	0.572
	T2	12.0 (1.8)	12.6 (1.6)	13.1 (0.9)	11.7 (1.9)	11.1 (1.9)	0.018
SPPB Mean (SD)	T1	9.5 (3.2)	10.2 (2.1)	10.4 (2.6)	8.2 (4.1)	10.2 (2.2)	0.039
	T2	8.6 (3.3)	9.7 (2.6)	9.8 (2.3)	7.1 (4.2)	9.0 (2.2)	0.018
HGS Mean (SD)	T1	27.9 (9.6)	27.7 (8.6)	32.3 (9.1)	26.1 (11.3)	28.8 (7.3)	0.386
	T2	27.2 (8.6)	28.5 (8.4)	29.9 (8.9)	24.4 (9.2)	28.8 (6.9)	0.171
WC Mean (SD)	T1	100.2 (10.5)	99.5 (10.1)	98.6 (11.5)	100.2 (12.0)	102.3 (7.9)	0.822
	T2	100.9 (10.7)	99 (7.7)	98.1 (12.3)	101.7 (13.6)	104.4 (6.6)	0.382
Length of stay in the UK		43.3 (16.3)	51.2 (10.4)	43.8 (17.9)	34.5 (17.6)	46.1 (14.4)	0.001
mean (SD) years							

*SD* Standard deviation; *MNA-SF* Mini Nutritional Assessment-Short Form; *SPPB* Short Physical Performance Battery; *HGS* Handgrip strength; *WC* Waist circumference

* Mean difference between changes in social network groups. (*P* value calculated using chi-square and Kruskal Wallis tests where appropriate)

One-way analysis of variance was conducted to ascertain the difference in energy and nutrient intakes between the changes in social network groups at follow-up. The groups differed in the intakes of folate, potassium, sodium, and percentage total energy (%TE) from saturated fat. Compared to those who changed to the non-integrated network group, those who remained in the integrated network group had lower intakes of %TE from saturated fat (*P* = 0.009) that were within the UK Recommended Nutrient Intakes (RNI) for %TE saturated fat. Similarly, those who remained in the integrated network group had significantly higher intakes of folate (133.3 μg.day^− 1^) as compared to those who changed to the non-integrated network group (94.8 μg.day^− 1^) (*P* = 0.045). Potassium intakes also differed significantly between the groups (*p* = 0.015). Those who changed to the integrated network group had higher intakes (1959.5 mg.day^− 1^) as compared to those who changed to the non-integrated network group (1412.1 mg.day^− 1^) and who remained in the non-integrated network group (1501.2 mg.day^− 1^) (*P* = 0.015). As compared with the age specific UK RNI for folate, 200 μg.day^− 1^, and potassium 3500 mg.day^− 1^, the intakes of folate and potassium for all changes in social network groups were below the recommended intakes. Sodium intakes for all groups were also below the UK RNI of 2400 mg.day^− 1^. However, those who changed to the integrated network group had significantly higher intakes of sodium (1536 mg.day^− 1^) as compared to those who changed to the non-integrated network group (895.3 mg.day^− 1^) and those who remained in the integrated network group (1006.3 mg.day^− 1^) [see Additional file 2 for details].

### Perceived social changes and reasons for social network changes

Almost one-third (28%) of participants reported perceived changes in their social networks during follow-up. Of those perceiving changes, the quantitative data supported these changes in 18 (22%), indicating changes to non-integrated or integrated social network groups, as shown in Table 3. The majority (12; 67%) of those that changed to the non-integrated network, reported perceived declines in their social networks. These participants cited changes in living arrangements, and reduced frequency of contact from children, other relatives and friends as the main reasons for the decline in network size and density. For instance, a woman living alone and belonging to the ‘changed to the non-integrated social network group’ at follow-up attributed the reduction in network size to the sudden, infrequent visits from her children as compared to baseline:

*“No, there’s no increases [in social networks], it has decreased since we met. I don’t know why, but my children … . because some of them [children], when they have the partner, the partner doesn’t like them to come and visit mum, I said to them it’s up to you, don’t let anybody tell you, “oh you can’t go by your mum”. You should go by your mother, your mother gave birth to you, but I can’t force someone to come … (P72, 65-69 years).*

Another participant explained that her network size had stayed the same over the study period, however, the frequency of contact of network members (family and friends) had reduced.

*“The network has stayed the same, but the frequency of contact has reduced” (P100, 60-64 years).*

Participants listed Illnesses that made them less mobile as one reason for experiencing declines in their social networks. The decrease in physical function resulting from illness prevented them from visiting their social groups as regularly as they used to. A participant recounted how his ill-health during the study period kept him housebound, hence the reason she no longer sees some of her friends.

*“Yes, because I’m getting more immobile in my legs. My hip and my legs, I don’t know what is causing it, maybe the sickness in this couple of months … .that has made me not able to go there again [community centre] … I don’t see my friends again as I used to” (P77, 65-69 years).*

Participants who remained in the non-integrated social network at follow-up described no improvement in their social networks. In some cases, participants described their social networks as poorer than before. For instance, a male participant reported that he is alone most of the time, and the few friends that he used to spend time with are unwell.

*“It is the same if not bad now, I don’t have my family here as I said, I am alone here … … . I have a few friends that I used to see from time to time, they are sick now, I am by myself most times” (P18, 60-64 years).*

Participants that remained in the integrated group were more likely to describe a stronger social network, attributing it to the increase in family network size, for example, the presence of grandchildren or the relocation of their children back to Birmingham. As explained by a male participant living with his spouse and youngest daughter, he outlines how his network size has improved over the study period.

*“Oh, the size is increasing because the other one [son] has got two children now. Yeah, and the relationship is getting even tighter because the children come here, their mum comes here almost every day. So, the relationship is tight” (P37, 60-64 years).*

### Associations of social networks with physical function, nutrient intake and nutritional status at baseline

Hierarchical multiple linear regressions were performed to determine the relationship of social network types with physical function (SPPB), nutritional status, BMI, waist circumference and nutrient intakes. Table 4 shows statistically significant predictors of fully adjusted models for the outcome variables: SPPB, zinc, riboflavin, vitamin B6 and manganese. Models of social networks and other independent variables predicting BMI, waist circumference, handgrip strength, nutritional status and all other nutrients as outcome variables were not statistically significant [See Additional file 3].

**Table 4:** Hierarchical multiple linear regression predicting SPPB, zinc, riboflavin, vitamin B6, manganese and folate intakes at baseline(*N* = 100)

	SPPB		Zinc		Riboflavin		Vitamin B6		Manganese	
Sociodemographic variables	B (95%CI)	SE	B (95%CI)	SE	B (95%CI)	SE	B (95%CI)	SE	B (95%CI)	SE
(Constant)	17.33 (10.8, 23.85)**	3.28	1.01 (−3.45, 5.47)	2.25	−0.34 (−1.54, 0.85)	0.60	0.09 (−1.07, 1.24)	0.58	0.99 (−2.03, 4.01)	1.52
Sex	−0.36 (− 1.50, 0.78)	0.57	0.26 (− 0.51,1.04)	0.39	0.12 (− 0.09, 0.33)	0.10	0.10 (− 0.11, 0.30)	0.10	− 0.04 (− 0.57, 0.48)	0.27
Age	− 0.09 (− 0.16, − 0.02)*	0.03	− 0.02 (− 0.03, 0.06)	0.02	0.10 (− 0.01, 0.02)	0.01	0.01(− 0.01, 0.01)	0.01	0.01 (− 0.02, 0.05)	0.02
IMD	0.05 (− 0.23, 0.32)	0.14	0.01 (− 0.19, 0.18)	0.09	0.10 (− 0.05, 0.05)	0.03	−0.03 (− 0.08, 0.02)	0.02	− 0.04 (− 0.16, 0.09)	0.06
Education	− 0.09 (− 0.60, 0.43)	0.26	0.10 (− 0.25, 0.46)	0.18	0.16 (0.06, 0.25)**	0.05	0.17 (0.07,0.26)**	0.05	0.11 (−0.13, 0.35)	0.12
Self-rated health	−1.57 (−2.29, −0.84)*	0.37	0.11(−0.39, 0.61)	0.25	0.04 (−0.09, 0.18)	0.07	0.01 (−0.14, 0.12)	0.07	0.10 (−0.24, 0.44)	0.17
Number of diseases	−0.10 (− 0.57, 0.37)	0.24	0.31 (− 0.01, 0.63)	0.16	0.04 (− 0.05, 0.12)	0.04	0.03 (− 0.05, 0.12)	0.04	− 0.12 (− 0.33, 1.36)	0.11
**Social networks**										
Locally integrated	3.06 (0.69, 5.44)*	1.20	1.29 (−0.33, 2.92)	0.82	0.14 (−0.30, 0.57)	0.22	0.16 (−0.29, 0.59)	0.21	0.26 (−0.84, 1.36)	0.55
Wider community	3.47 (0.45, 6.48)*	1.52	1.72 (−0.35,3.78)	1.04	0.12 (−0.43, 0.68)	0.28	0.13 (−0.40, 0.67)	0.27	1.67 (0.27, 3.06)*	0.70
Family dependent	2.97 (0.49, 5.45)*	1.25	0.56 (−1.14, 2.26)	0.85	0.07 (−0.38, 0.53)	0.23	0.22 (−0.22, 0.66)	0.22	−0.23 (−1.38, 0.92)	0.58
Local self-contained	2.25 (−0.30, 4.81)	1.29	2.27 (0.52, 4.02)*	0.88	0.50 (0.03, 0.96)*	0.24	0.54 (0.09, 0.99)*	0.23	0.38 (−0.81, 1.56)	0.60
Private restricted	Ref		Ref		Ref		Ref		Ref	
*R*^*2*^	0.365		0.205		0.231		0.231		0.186	
*R*^*2*^*change*	0.055		0.101		0.080		0.086		0.114	
*F for change*	1.942		2.829		2.324		2.477*		3.131*	

*SPPB* Short Physical Performance Battery; Sex was coded as male = 1, female = 2; Education was coded no education, primary = 2, secondary = 3, college/university = 4; self-rated health was coded as excellent = 1, good = 2, fair = 3 and poor = 4. IMD = Index of multiple deprivation. 1 is least deprived and 4 is most deprived

*B* Unstandardized regression coefficient; *SE* Standard error of the coefficient; *CI* Confidence interval; *P value < 0.05; **P value < 0.01

With respect to physical function (as indicated by SPPB), the fully adjusted model shows that age, sex, IMD, self-rated health, number of diseases, education and social networks were significant predictors [F (10, 89) = 5.11, *p* < 0.001], contributing to 36.5% of the variance in SPPB scores. Older adults who belonged to the integrated network, wider community network and family-dependent social networks had SPPB scores that were 3.06, 3.47 and 2.97 points higher, respectively, than those in private restricted network (the reference group), social networks explained an additional of 5.5% of the variance in SPPB. Also, within the fully adjusted model, participants who were older and who self-reported poorer health were more likely to have lower SPPB scores than the younger old with good self-reported health.

For zinc, the fully adjusted model was significant [F(10, 89) = 2.302, *p* = 0.19], and the variables age, sex, IMD, self-rated health, number of diseases, education and social networks explained 20.5% of the variance of zinc. Participants belonging to the local self-contained network reported a 2.27 mg/day higher zinc intake as compared to participants in private restricted network, and this contributed to an additional 10.15% of the variance of zinc intake. Similarly, for riboflavin intake, the model was significant, [F (10, 89) = 2.668, *p* = 0.07 and the variables age, sex, IMD, self-rated health, education and social networks contributed a total of 23.1% of the variance of riboflavin intake. Older adults belonging to the local self-contained network reported a 0.51 mg/day higher riboflavin intake than the private restricted network, explaining 8.6% of the variance in riboflavin intake. A similar result was found for the fully adjusted model for vitamin B6 [F(10, 89) = 2.67, *p* = 0.007], with age, sex, IMD, self-rated health, number of diseases, education and social networks accounting for 23.1% of the variance in vitamin B6 intake. Older adults belonging to the local self-contained network as compared to the private restricted network consumed 0.54 mg/day more vitamin B6, accounting for an additional 8.6% of the variance.

For manganese intake, results differed in that the fully adjusted model was significant, [F(10, 89) = 2.04, *p* = 0.038], and the variables age, sex, IMD, self-rated health, number of diseases, education and social networks explained 18.6% of the variance of manganese intake. Older adults belonging to the wider community network had 1.64 mg/day higher intakes of manganese than the private restricted network, and this explained an additional of 11.4% of the variance of manganese intake.

### Perceptions of the impact of social networks on eating behaviours and physical function at baseline

It emerged that some participants (28%) perceived their current social networks ties to be influencing their physical function and eating behaviours. Many (67%) of them expressed the changes in living arrangements, especially the absence of children in the household as compared to baseline, to be the key influence on their eating behaviours.

*“As the children, all moved out, my diet has changed, yes, at that time, you know, there was more salad, and yes, I usually did a little bit more extra … there was always more food, so I ate more may be better but now...[shaking her head] no” (P19, 80-84 years).*

*“Um, I wouldn’t say my eating habits have improved, because like when the children were at home, we used to have a lot of eating times, but now I think it’s reduced cos we are alone now. Also, I tend to be eating more now (laughs), because the excuse is, there’s more to eat, and I don’t wanna waste it (laughs)” (P24, 80-84 years).*

A few (15%) also identified that changes in living arrangements and social networks impacted on their physical function. They felt that the presence of the children or other social ties served as motivation to be more physically active, as they used to do activities together.

*“Well, we used to have exercises a lot when they [children] were home, you know, especially when my daughter was at home because I used to have an exercise bike that we shared together. I still got a few exercise equipment thrown in the cupboard in there, no motivation to bring them out” (P28, 80–84 years).*

The social support received during commensality was mixed, with the majority (62%) of males living with spouses preferring it, describing it as helping them to eat more healthily. Even though females also preferred the act of commensality, some said it led them to consume larger portion sizes than they would normally eat. However, a few women (6%) said it had no influence on their portion sizes. These views are supported by the quotes presented below:

*“Yeah, my, my wife supports me to eat healthily. Always reminding you about what you have to eat especially when we eat together and even the times to eat and then, with exercises, exercise also, she says “Oh, I haven't seen you step on this treadmill for some time.” she will [be] saying it till you do it (laughs)” (P30, 60-64 years).*

*“He’s [Husband] always telling me that I’m not eating enough carbohydrates, like this morning he decided he was going to do egg and toast for us, and you should have seen the slice of the bread he cut! … … Yes, it does influence as sometimes I tend to eat too much, just like this morning” (P63, 60-64 years).*

Five percent of men living with their spouse preferred to eat alone and explained that eating alone allowed them to eat what and when they wanted, without interference from their spouse or other family members. As explained by a 60-year-old man:

*“My wife is the one who jumps up and about, “Shouldn’t be eating this, this is not good.” I don’t like it … I stubbornly still put it [the food] on my plate regardless and I say, “Look, I’m going to die one day, let me die in style, at least you can say I went after eating what I enjoyed best (laughs)” (P14, 60-64 years).*

### The association of social networks on physical function (SPPB) and nutritional status (MNA-SF) over time

Multiple linear regression was conducted to ascertain the impact of baseline social networks on physical function (SPPB) and nutritional status at follow-up. These analyses were controlled for sex, age, IMD, self-reported health, educational status and baseline measurement of SPPB or MNA-SF depending on the outcome variable. As shown in Table 5, the fully adjusted model shows that age and baseline SPPB were significant predictors [F (7, 73) = 40.7, *p* < 0.001] of SPPB scores at follow-up, contributing to 77.8% of the variance in follow-up SPPB scores. Participants who belonged to the integrated SN had SPPB scores that were 0.88 points higher than those in non-integrated SN (the reference group); SN explained an additional of 1.5% of the variance in SPPB.

**Table 5:** Social network and socio-demographic variables association with physical function and nutritional status at follow-up (N = 81)

	SPPB at follow-up				MNA-SF at follow-up			
Sociodemographic variables	B (95%CI)	SE	Standardized Coefficients Beta	***P***-value	B (95%CI)	SE	Standardized Coefficients Beta	***P***-value
(Constant)	5.84 (1.07, 10.6)	2.39		0.02	6.49 (1.19, 11.80)	2.66		0.01
Sex	0.24 (−0.49, 0.98)	0.37	0.03	0.52	−0.50 (−1.25, 0.25)	0.38	−0.13	0.19
Age	−0.07 (− 0.12, − 0.03)	0.02	−0.18	0.00	−0.01 (− 0.06, 0.03)	0.02	−0.06	0.55
IMD	0.17 (−0.01, 0.36)	0.09	0.10	0.06	0.001 (−0.19, 0.18)	0.09	0.00	0.99
Education	−0.01 (− 0.36, 0.33)	0.17	− 0.01	0.93	0.14 (− 0.20, 0.49)	0.17	0.09	0.42
Baseline SPPB/MNA-SF*	0.79 (0.65, 0.93)	0.07	0.74	0.00	0.47 (0.23, 0.71)	0.12	0.42	0.00
Self-reported Health	−0.31 (−0.79, 0.16)	0.24	−0.08	0.19	0.21 (−0.23, 0.65)	0.22	0.10	0.35
**Social networks**								
Integrated	0.88 (0.12, 1.64)	0.38	0.13	0.02	0.97 (0.22, 1.72)	0.38	0.26	0.01
Non-integrated	Ref				Ref			
*R*^*2*^	0.778				0.221			
*R*^*2*^*change*	0.015				0.066			
*F for change*	5.396				6.727			

*SPPB* Short Physical Performance Battery; *MNA-SF* Mini Nutritional Assessment-Short Form; *For the adjusted model of follow-up SPPB as outcome variable, the model was adjusted for baseline SPPB, For the adjusted model of follow-up MNA-SF outcome variable, the model was adjusted for baseline MNA-SF. Sex was coded as male = 1, female = 2; Education was coded no education, primary = 2, secondary = 3, college/university = 4; self-rated health was coded as excellent = 1, good = 2, fair = 3 and poor = 4. *IMD* Index of multiple deprivation. 1 is least deprived and 4 is most deprived. *B* Unstandardized regression coefficient; *SE* Standard error of the coefficient; *CI* Confidence interval

Regarding nutritional status, the fully adjusted model as shown in Table 5 indicates that participants who were in integrated SN at baseline had MNA-SF scores that were 0.97 points higher at follow-up as compared to participants in non-integrated SN. SN contributed in explaining 6.6% of the variance in MNA-SF at follow-up. Also, baseline MNA-SF explained 22.1% of the variance of MNA-SF at follow-up. Other than baseline MNA-SF and SN, there were no significant predictors of MNA-SF at follow-up [F (7, 73) = 4.24, *p* = 0.001].

### Perceived influences of social network changes on eating behaviours and physical function

At follow-up, participants’ perceptions of the impact of changes in social networks on their eating behaviours and physical function were explored. Out of the 28% of participants perceiving changes to their social networks, men were more likely to describe these changes as unfavourable as compared to women. Most men (78%) highlighted the decline in social networks to be directly influencing their shopping, cooking and physical function. A male participant described the loss of his spouse during the study period as impacting on his eating behaviours. He highlighted that he now chooses to eat ‘ready to eat’ meals opposed to traditional meals, citing a lack of cooking skills and a lengthy amount of time required to cook his traditional foods.

*“Well, it [eating behaviours] has changed quite a lot, to be honest. Because as I said, I used to have my wife … my wife used to cook, but now I have to do all that myself and it is not easy mainly because it’s me, shopping it myself is not easy … . I don’t know how to cook it [traditional foods] myself and it takes long. So, I don’t eat them [traditional foods] like l used to … .I just go for the easy packaged ones [ready to-eat foods]” (P58, 65-69 years).*

Over the study period, participants that perceived a decline in social network attributed it as part of the reasons for a decrease in their regular physical activities, eventually leading to a reduction in physical function. One participant described how the recent decline in the frequency of meeting with her friends is making her “lazy” and less likely to engage in her regular physical activities as before.

*It [social networks] has reduced. E and Z used to come around. They are my only friends now. We used to visit the park or go for a meal. Things have changed, E is caring for her sick husband now, and for Z I don’t know what is wrong with her. If I don’t ring, she will not ring. Well, I don’t know much but I think that has probably made me a bit lazy … I don’t want to walk on my own (P74, 60-64 years).*

Contrary to participants expressing the decline in social networks influencing their eating behaviours and physical function, a few participants (12%) reported a perceived increase in social networks during follow-up. They described it to be positively influencing them to eat well and to maintain their physical function. A participant recounted that during the follow-up period, she had made new friends who motivated her to join cycling and other physical activity groups in the community.

*“More of an increase, really because I made new friends, this friend that came from London, she's like this [slim]. So, she'll say, "Come on, we're doing a bike ride." And she'll say, "Come on, we're going yoga." And she's very much active, because of her I have joined this evening group in the last eight months, that we all do this walking” (P68, 60-64 years).*

Within this group expressing improvement in social networks at follow-up, some (43%) discussed how the improvement of social networks was linked to some improvements in their eating behaviours, as expressed by a female participant below:

*“Friends are maybe more now as compared to before, and when I’m saying ‘friends,’ I’m thinking about church, especially my local church. We eat a lot at church this time [within the 8-months] and again, sometimes there is a swapping of ideas around food” (P51, 70-74 years).*

Regular engagement in religious and community group meetings were perceived to be influencing eating behaviours and physical function. The majority (68%) of participants attending these group meetings highlighted the importance of these regular meetings, stating that it allowed them to meet with friends, share food and other healthy eating tips. These participants (68%) also cited the regular group exercises at some of these meeting centres to be important in maintaining their physical function, as explained by a female participant below:

*“Well coming here [community centre], you get to [do] your exercise, and then you can see your friends as well, that’s very important. This motivates me to come. I mean, otherwise, you won’t see them. And they keep your mind occupied as well. And you do, like, the exercise, you lose one stone or two, you become fitter, it’s like killing two birds with one stone” (P88, 70-74 years).*

## Discussion

This is the first study to explore changes in social networks, and their association with nutritional status, nutrient intake and objectively measured physical function monitored longitudinally among community-dwelling ethnically diverse older adults in the UK. Considering the growing population of ethnic minorities in the UK and the existing health inequalities faced by these groups, the present findings form part of an essential process of advancing our understanding of social network dynamics and their implications for a healthier ageing trajectory in this population [12, 63]. A clearer understanding of social networks could help design and implement culturally sensitive community interventions to improve social networks and promote healthy ageing.

We identified five social networks grouped under two major categories: 1) the integrated social network, consisting of the locally integrated social network and the wider community network; and 2) the non-integrated network, consisting of the family-dependent, the local self-contained and the private restricted networks. While most participants at baseline (45.7%) and follow-up (37.0%) belonged to the locally integrated social network, only a few at baseline (6.2%) and follow-up (7.4%) belonged to the private restricted social network. This is consistent with previous studies of social networks in the UK using similar network generation typologies [40, 56, 64]. Even though there seemed to be high proportions of non-integrated social networks among Caribbean and African participants, there were no significant differences in social networks between ethnicities as reported by previous studies in ethnic minority populations in the UK and elsewhere [22, 64]. This could have been heavily influenced by the recruitment process, as most recruitment was done in social gatherings that were ethnic specific. Additionally, given the convenience sampling and the use of techniques such as maximum variation sampling in this study, it is possible that these approaches introduced selection bias, which could explain observing no differences in social networks among ethnicities. However, social networks differed by sex; despite the high proportion of men being married, they had significantly higher proportion of restricted social networks than women. The present findings are not consistent with other studies which reported that older married men had more extensive networks as compared to women that were unmarried or widowed [65]. As explained by our qualitative findings, women (both married and widowed) expressed high community involvements with faith groups and other community activities, thus accounting for a higher proportion of integrated social networks than their male counterparts.

Furthermore, in contrast to previous findings, our findings indicated a small proportion of restricted social networks at baseline and follow-up (6.2 and 7.4%, respectively) [22, 64, 66]. One possible explanation could be the different social network typologies used, that is, the use of latent class, cluster analysis or name generation of network groups as compared with the Wenger PANT tool. For instance, a study of changes in social networks among older Koreans using latent class analysis to categorise older adults into groups found that at both study points, the most prevalent social network group was the restricted social network [62]. In addition, the recruitment strategy used in the present study could be accounting for these differences. Participants in the present study were recruited from community centres, faith groups and other social events, while other published studies analysed large national or international datasets of participants who were recruited using postal addresses, hospital registers or directly from national records [66, 67].

Despite the small number of participants reporting restricted social networks, the present study provides novel findings of the dynamics across social networks over time in ethnic minority older adults. Within a relatively brief follow-up period, quantitative findings indicated that 24 (29.6%) participants reported changes in social networks. As compared to many studies with longer follow-up periods, the finding of changes in social networks within 8 months is unique, providing valuable insights on the stability of social networks within this population even over brief periods. Even though the direction of change was not always towards the non-integrated social networks as anticipated, the majority of those that changed, 15 (62.5%), moved from an integrated social network to a non-integrated social network. This sharply contrasts with a study of social network changes among older Europeans, where over a 4-year period, Europeans aged 65 years and older experienced an expansion of network ties [67]. Explained through the qualitative results, participants within the present study noticed the changes to a more restrictive type, describing a reduction in network size and frequency being due to less interaction with their children and friends. A striking finding was that sex, age, marital status and IMD scores were not associated with changes in social network groups at follow-up, which contrasts with previous studies [66, 67]. For instance, a 2-year longitudinal study exploring social network changes among adults aged 60 years and older found that sex, age and self-rated health were significant predictors of changes in social networks at follow-up [65]. The authors of this previous study observed that participants who remained in the poorer social networks at follow-up were typically females, older, having poorer socio-economic status and self-rated health [65]. Also, unlike the present study, a recent 4-year longitudinal study of Europeans aged 65 years and older also found that women had relatively larger social networks at follow-up [67]. One possible explanation accounting for these differences in findings across studies could be the relatively small sample size (for quantitative analysis) and the convenience and purposive sampling of the present study as compared to the other studies. However, in the present study, social network changes did differ significantly by the length of stay in the UK, SPPB and MNA-SF scores. The maintained integrated group had been living in the UK longer than the changed or maintained non-integrated groups. Length of stay in the UK has been previously shown to vary among migrants in the UK, with Black Caribbean people showing the longest stay in the UK as compared to other ethnicities [22]. In the present study, it is likely that the increased length of stay enabled older adults to form stronger and more stable integrated networks as compared to those with shorter stays. This is supported by studies elsewhere that found longer residence in the UK was associated with more stable integrated social networks than in those with shorter stays [68, 69].

As explained by our qualitative findings, good health in terms of stronger physical function and better nutritional status was essential to maintaining a stronger social network. This evidence supports the theory of constraint of social networks, which explains that structural constraints such as a decline in physical health and other changes associated with ageing cause a decline in personal social networks. Hence, it was not surprising that participants remaining in, or transitioning to, integrated networks had significantly higher SPPB and MNA-SF scores than the participants who were in, or who transitioned to, non-integrated social networks. Nevertheless, given the limited follow-up period of 8 months, further investigations are required to confirm these factors associated with declines of social networks within this population over time.

### The association of social networks and health outcomes

The findings of the present study indicated that the locally integrated social network was associated with increased SPPB scores when compared to the private restricted network, even after adjusting for age, sex, IMD, self-rated health, number of diseases and education. During the qualitative interviews, participants described poor social networks at baseline to be affecting their eating behaviours and physical function. As expected and consistent with previous findings, at follow-up, participants who remained in, or transitioned into, non-integrated networks had significantly lower physical function scores as compared to those that maintained, or transitioned to, integrated social networks [70]. In the adjusted model, belonging to a non-integrated social network at baseline and increased age were associated with lower physical function at follow-up. Similarly, it was observed that participants belonging to integrated SN at baseline had higher MNA-SF scores at follow-up as compared to those who were in non-integrated SN at baseline. Even though SN was a significant predictor of physical function or nutritional status at follow-up, SN explained a relatively small percentage of the variance of these outcome variables. One possible contributor to these findings is the length of follow-up, as an 8-month follow-up period might not be long enough to observe a greater impact of changes in participants’ SN on their physical function and nutritional status. For instance, from the qualitative findings, participants perceiving declines in SN at follow-up mentioned the declines in frequency and size of their SN to be contributing to poorer physical function and eating behaviours. The detrimental impact of this sudden change in lifestyle behaviour on nutritional status and physical function might take longer to assess using the tools employed in the current study. Despite this, the findings from this longitudinal mixed methods study suggest that integrated social networks may be protective against declines in physical function and nutritional status declines over time among community-dwelling ethnically diverse older adults. The protective effect of large social networks on physical function has also been reported in predominately White populations, with similar profile of non-communicable diseases [71]. Considering the age-related decline in physical function, our data suggest that interventions and public health strategies aimed at supporting older adults to maintain an integrated SN may have the potential to prevent declines in physical function and nutritional status, and promote healthy ageing.

In the UK, ethnic minorities report lower self-rated health as compared to the rest of the population [72]. For instance, in the 2004 Health Survey for England (HSE), 15% of Bangladeshi men, 14% of Bangladeshi women, 10% of Pakistani men, and 15% of Pakistani women reported poor health as compared to the 6% men and 7% women in general population [72]. In the present study, there were no differences in self-rated health among ethnic minorities, with approximately one-third reporting fair or poor health. However, self-related health differed by social networks. We found that social networks and changes in social networks were significantly associated with self-rated health. Older ethnic minorities belonging to the non-integrated social networks, as compared to integrated social networks, reported significantly higher proportions of poor or fair health. Similarly, participants that transitioned from an integrated social network to a non-integrated social network were more likely to report fair or poor health as compared to the maintained integrated group. These findings are consistent with studies in indigenous populations reported elsewhere [73].

Findings from the present study are consistent with the model of ‘social control’ on healthy behaviours in old age [74]. Participants with integrated social networks recounted the importance of their spouse and the wider relationships on their eating behaviours and physical function. The findings enlighten the direct path of ‘social control’ played by their spouses in either providing healthy food or healthy eating messages as described in the ‘social control’ model [74]. Outside the family, some participants that attended community centres or other faith groups saw it as a valuable contribution to improving their eating behaviours and physical function. As previously reported [75], the activities of these centres provide at least one of the three broad components of social support: informational (the dietary advice received); emotional (providing a sense of empathy); and instrumental support (exercise classes or free medical assessment received). These findings further broaden understanding of the mechanism related to how increased social ties might impact the health of older adults. The benefits of these social groups as purported in this present study have also been reported by older Asian adults living in the United States [23]. Anecdotally, it was observed that even though these centres provided this level of support, most of these centres were either closed or reduced their opening days during the study period due to budget cuts, threatening the sustainability of these support groups.

The lack of statistically significant differences in nutrient intakes, except fibre intake, between the most (integrated SN) and least robust (private restricted SN) social network groups within this study was unexpected and in contrast to existing literature showing that more robust social networks were associated with healthier nutrient intake [76, 77]. As previously stated, the smaller sample size, especially for the private restricted social network group, and the convenience and purposive sampling techniques used could have influenced these results. However, the two least robust social networks (local self-contained compared with private restricted) were found to be significant predictors of zinc, riboflavin and vitamin B6 intakes. These two non-integrated networks are defined by having relatively reduced frequency of contact and the absence of local kin [37, 43]. Additionally, participants in the present study reported less company during meals and minimal or no influence of family and friends during mealtimes. One possible explanation for this unexpected finding could be the higher intake of nutrient supplements reported among older adults in the local self-contained social network (61%) as compared to the private restricted social network (16.7%).

Intakes of sodium, potassium, folate and %TE from saturated fat differed significantly by changes in social network groups at follow-up. Older adults who remained in, or transitioned to, the integrated network had significantly higher intakes of these nutrients (except sodium). Despite the consumption of potassium and folate being below the UK RNI, these older adults within the integrated networks could have been influenced by the ‘informational support’ at their regular community meetings and even at home as evidenced by the qualitative findings. Even though awareness does not necessarily translate into behaviour change, it is possible that older adults attending these meetings that offer dietary advice and many times provide meals benefit from this attendance, which could contribute to the difference in nutrient intakes observed. Given the relatively small sample size (in relation to the quantitative analyses) and limited follow-up time of 8 months, these findings should be viewed with caution, and we recommend that future studies should be designed with a longer follow-up period to carefully examine these findings.

### Strengths and limitations of the study

The study was conducted in an under-represented research population which is a significant strength. Additionally, the longitudinal mixed methods element made it possible to explore the changes in social networks, the participants’ perspectives of their social networks, and the influence of these changes of social networks on physical function and nutritional status. The sample size, although relatively small from a quantitative perspective, was large for a qualitative study. Compared to previous studies using subjective measure of physical function, the inclusion of objectively measured physical function (SPPB and handgrip test) provided a more accurate representation of the relationship between social networks and physical function in the present study. However, this study had limitations. Firstly, by design, the 8 months duration of follow-up was relatively short. This length of time was chosen due to the practicality of the data collection within the timeframe of the project. Secondly, although the study employed a maximum variation sampling technique to ensure a wider representation of the sample, it was difficult to recruit South Asian women, particularly women of Bangladeshi origin. Thirdly, this study shares a common limitation encountered within many studies exploring dietary intake, which is the inherent limitations of self-reported nutrient intake. Even though measures were put in place to enhance accurate self-recalls, it is still possible that some participants under-reported or over-reported their nutrient intakes. Lastly, as most of the recruitment was done through social gatherings, this made it difficult to recruit highly socially isolated participants. This could have accounted for the high representation of integrated social networks and the no significance in social networks observed among ethnicities. It is recommended that future studies employ various recruitment approaches that can enhance the recruitment of more isolated participants across diverse populations of older ethnic minorities.

## Conclusion

This longitudinal mixed methods study produced unique findings regarding the changes in social networks among community-dwelling ethnic minority older adults over 8 months. Even though the direction of change was not always towards the non-integrated social networks as expected, the majority moved from integrated to non-integrated social networks. Overall, the findings indicated that integrated social networks at baseline were protective against declines in physical function and nutritional status at follow-up and were associated with better self-rated health. These findings further demonstrate the important role of stronger social networks in healthy ageing, and the need to develop community-based interventions to support older ethnic minorities embedded in restricted social networks to age more healthfully. Given the benefits of social support derived from attending community groups found in this study, improving the access of these centres and providing long-term financial support from interested organisations and public funding sources could improve the sustainability of these ethnic and religious centres. This could, in turn, support the maintenance of integrated social networks among community-dwelling older adults. Additionally, the findings of this study could be used to tailor community interventions to make them more culturally sensitive to support community-dwelling older adults to age more healthily. Future studies with an extended follow-up and larger sample sizes within this population are recommended to further elucidate the temporality and quality of changes in social networks and their relationship with dietary intake and changes in physical function and nutritional status in this population.

## Supplementary information

**Additional file 1.** Demographic characteristics and other variables by the broad two social networks.

**Additional file 2. **Social network changes and nutrient intakes among community-dwelling ethnically diverse older adults (*n*=81)

**Additional file 3.** Association of social networks with MNA-SF, WC, HGS and selected nutrients.

## Data Availability

The datasets generated and analysed during the current study are part of a larger PANINI network data set containing unpublished data, hence are not currently available. The data will be made available after completion of the Innovative Training Network (ITN) project.

## Abbreviations

SN: Social Networks
UK: United Kingdom
US: United States of America
CI: Confidence interval
SD: Standard Deviation
IMD: Index of Multiple Deprivation
MNA-SF: Mini Nutritional Assessment-Short Form
SPPB: Short Physical Performance Battery
HGS: Handgrip strength
BMI: Body Mass Index
