# Supplementary material for: Social networks and their influences on nutrient intake, nutritional status and physical function in community-dwelling ethnically diverse older adults: a mixed-methods longitudinal study
Source: BMC Public Health. 2020 Jun 26;20:1011. doi: 10.1186/s12889-020-09153-y (PMC7318427; doi:10.1186/s12889-020-09153-y)
Supplement: Supplementary file 1 — Additional file 1. Demographic characteristics and other variables by the broad two social networks. [file 12889_2020_9153_MOESM1_ESM.docx]

**Additional files**

| **Variable** | | **Integrated SN (n=52)** | **Non-integrated SN (n=48)** | **P-value^α^** |
| --- | --- | --- | --- | --- |
| **Age Mean (SD)** | | 71 (8.0) | 71 (7.7) | 0.495 |
| **Sex N (%)** | Male | 31 (60.0) | 28 (58.3) | 0.897 |
| **Marital Status^¥^** | Married | 39 (75.0) | 27 (56.3) | 0.049 |
|  | Not married | 13 (25.0) | 21 (43.8) |  |
| **Ethnicity N (%)** | Caribbean | 19 (36.5) | 22 (45.8) | 0.254 |
|  | Pakistani | 15 (28.8) | 8 (16.7) |  |
|  | African | 8 (15.4) | 11 (22.9) |  |
|  | Indian | 4 (7.7) | 3 (6.3) |  |
|  | Bangladeshi | 2 (3.8) | 2 (4.2) |  |
|  | Others**^β^** | 4 (7.8) | 2 (4.2) |  |
| **Faith‎/Religion N (%)** | Christian | 26 (50.0) | 30 (62.5) | 0.246 |
|  | Muslim | 21 (40.0) | 13 (27.1) |  |
|  | Sikh | 4 (7.7) | 3 (6.3) |  |
|  | Hindu | 1 (1.9) | 1 (2.1) |  |
|  | No religion | 0 (0.0) | 1 (2.1) |  |
| **Self-R health N (%)** | Excellent/good | 39 (75.0) | 24 (50.0) | 0.010 |
|  | Fair/poor | 13 (25.0) | 24 (50.0) |  |
| **IMD Quartiles N (%)** | 1 (Most deprived) | 18 (34.6) | 15 (31.3) | 0.802 |
|  | 2 | 8 (15.4) | 11 (22.9) |  |
|  | 3 | 11 (21.2) | 11 (22.9) |  |
|  | 4 (least deprived) | 15 (28.8) | 11 (29.9) |  |
| **SPPB Mean (SD)** | | 10 (3.0) | 9 (4.0) | 0.132 |
| **HGS Mean (SD)** | | 27.1 (8.5) | 26.5 (10.5) | 0.759 |
| **MNA-SF Mean (SD)** | | 12 (2.0) | 13 (2.0) | 0.751 |
| **BMI categories* N (%)** | Underweight | 0 (0.00) | 0 (0.0) | 0.702 |
|  | Normal | 3 (5.8) | 4 (8.3) |  |
|  | overweight | 16 (30.8) | 15 (31.3) |  |
|  | Obese | 33 (63.5) | 29 (60.4) |  |
| **Diseases n (%)** | Type 2 Diabetes | 23 (44.2) | 28 (58.3) | 0.160 |
|  | CVD | 11 (21.2) | 9 (18.8) | 0.755 |
|  | High BP | 25 (49.0) | 28 (59.6) | 0.274 |
|  | High Cholesterol | 6 (11.5) | 7 (14.6) | 0.635 |
|  | Cancer^µ^ | 5 (9.6) | 4 (8.3) | 0.824 |
|  | Others^∞^ | 6 (11.5) | 7 (14.6) | 0.635 |
| **Living alone %** | | 9 (17.3) | 16 (33.3) | 0.061 |
| **Supplement use** | Yes | 19 (38.5) | 19 (39.6) | 0.824 |
|  | No | 32 (61.5) | 29 (60.4) |  |

**Additional table 1: Demographic characteristics and other variables by the broad two social networks at baseline (N=100)**

SD= Standard deviation; IMD= Index of Multiple Deprivation; MNA-SF= Mini-Nutritional Assessment-Short Form; SPPB= Short Physical Performance Battery HGS= Handgrip strength; Self-rated health was coded as excellent=1, good=2, fair=3 and poor= 4. These further categorised as 1=Excellent/Good and 2=Fair/Poor; BMI= Body Mass Index

**Additional file 2: Social network changes and nutrient intakes among community-dwelling ethnically diverse older adults (n=81)**

| **Variable*** | **Changed to integrated (n=27)** | **Maintained integrated (n=9)** | **Maintained non-integrated (N=30)** | **Changed to non-integrated (N=15)** | **P value** |
| --- | --- | --- | --- | --- | --- |
| Energy Mean (SD) | 1609.4 (850.6) | 1543.8 (468.0) | 1914.4 (833.3) | 1777.3 (533.5) | 0.241 |
| %TE saturated fat Mean (SD) | 11.2 (3.8) | 9.8 (3.3) | 11.2 (4.3) | 14.0 (4.7) | 0.019 |
| Potassium mg.d^-1^ Mean (SD) | 1959.5 (501.2) | 1709.2 (548.3) | 1501.2 (406.9) | 1412.1 (288.3) | 0.015 |
| Sodium mg.d^-1^ Mean (SD) | 1536.3 (635.6) | 1006.1 (384.6) | 1095.8 (656) | 895.3 (298.7) | 0.030 |
| Folate µg. d^-1^ Mean (SD) | 140.3 (58.4) | 133.3 (45.2) | 115.3 (44.9) | 94.8 (33.6) | 0.032 |

***** showing only nutrients with statistically significant differences (except energy).

TE= Total energy

**Additional file 3: Association of social networks with MNA-SF, WC, HGS and selected nutrients.**

|  | MNA-SF | | | | | | | |
| --- | --- | --- | --- | --- | --- | --- | --- | --- |
|  | Model 1  (Unadjusted model) | | | | Model 2 (Fully adjusted model) | | | |
|  | B | SE | β | p value | B | SE | β | p value |
| (Constant) | 12.20 | 0.76 |  | 0.00 | 16.79 | 2.35 |  | 0.00 |
| Locally integrated | 0.38 | 0.81 | 0.12 | 0.64 | -0.48 | 0.84 | -0.15 | 0.57 |
| Wider community | 0.40 | 1.07 | 0.06 | 0.71 | 0.62 | 1.10 | 0.09 | 0.58 |
| Family-dependent | 0.47 | 0.84 | 0.12 | 0.58 | -0.21 | 0.86 | -0.06 | 0.81 |
| Local self-contained | 0.23 | 0.88 | 0.05 | 0.80 | -0.31 | 0.89 | -0.07 | 0.72 |
|  | Handgrip | | | | | | | |
|  | Model 1 (Unadjusted model) | | | | Model 2 (Fully adjusted model) | | | |
|  | B | SE | β | p value | B | SE | β | p value |
| (Constant) | 25.24 | 4.22 |  | 0.00 | 82.68 | 10.92 |  | 0.00 |
| Locally integrated | 3.63 | 4.51 | 0.19 | 0.42 | -4.06 | 3.91 | -0.21 | 0.30 |
| Wider community | -2.26 | 5.97 | -0.06 | 0.71 | -0.06 | 5.12 | 0.00 | 0.99 |
| Family-dependent | 5.27 | 4.70 | 0.24 | 0.27 | -3.07 | 4.00 | -0.14 | 0.45 |
| Local self-contained | -1.27 | 4.92 | -0.05 | 0.80 | -5.29 | 4.12 | -0.21 | 0.20 |
|  | Waist circumference | | | | | | | |
|  | Model 1 (Unadjusted model) | | | | Model 2 (Fully adjusted model) | | | |
|  | B | SE | β | p value | B | SE | β | p value |
| (Constant) | 108.15 | 4.72 |  | 0.00 | 109.90 | 15.09 |  | 0.00 |
| Locally integrated | -8.26 | 5.04 | -0.39 | 0.11 | -5.19 | 5.40 | -0.25 | 0.34 |
| Wider community | -7.05 | 6.68 | -0.16 | 0.29 | -6.83 | 7.07 | -0.16 | 0.34 |
| Family-dependent | -9.78 | 5.25 | -0.41 | 0.07 | -8.48 | 5.53 | -0.36 | 0.13 |
| Local self-contained | -7.56 | 5.50 | -0.27 | 0.17 | -6.11 | 5.70 | -0.22 | 0.29 |
|  | Energy kcal | | | | | | | |
|  | Model 1 (Unadjusted model) | | | | Model 2 (Fully adjusted model) | | | |
|  | B | SE | β | p value | B | SE | β | p value |
| (Constant) | 1902.60 | 233.72 |  | 0.00 | 2913.00 | 713.97 |  | 0.00 |
| Locally integrated | -173.09 | 249.43 | -0.17 | 0.49 | -213.18 | 255.67 | -0.21 | 0.41 |
| Wider community | -240.30 | 330.53 | -0.11 | 0.47 | -231.26 | 334.58 | -0.11 | 0.49 |
| Family-dependent | -74.70 | 260.06 | -0.06 | 0.78 | -221.52 | 261.80 | -0.19 | 0.40 |
| Local self-contained | -280.81 | 272.28 | -0.21 | 0.31 | -262.92 | 269.56 | -0.19 | 0.33 |
|  | Carbohydrates g | | | | | | | |
|  | Model 1 (Unadjusted model) | | | | Model 2 (Fully adjusted model) | | | |
|  | B | SE | β | p value | B | SE | β | p value |
| (Constant) | 229.65 | 28.64 |  | 0.00 | 319.08 | 88.19 |  | 0.00 |
| Locally integrated | -7.24 | 30.56 | -0.06 | 0.81 | -22.67 | 31.58 | -0.18 | 0.48 |
| Wider community | -24.11 | 40.50 | -0.09 | 0.55 | -28.28 | 41.33 | -0.11 | 0.50 |
| Family-dependent | -7.20 | 31.86 | -0.05 | 0.82 | -30.82 | 32.34 | -0.21 | 0.34 |
| Local self-contained | -36.03 | 33.36 | -0.22 | 0.28 | -44.34 | 33.30 | -0.27 | 0.19 |
|  | Fibre g | | | | | | | |
|  | Model 1 (Unadjusted model) | | | | Model 2 (Fully adjusted model) | | | |
|  | B | SE | β | p value | B | SE | β | p value |
| (Constant) | 20.91 | 3.40 |  | 0.00 | 20.23 | 9.36 |  | 0.03 |
| Locally integrated | -3.39 | 3.62 | -0.23 | 0.35 | -6.49 | 3.35 | -0.43 | 0.06 |
| Wider community | 0.77 | 4.80 | 0.03 | 0.87 | -1.87 | 4.39 | -0.06 | 0.67 |
| Family-dependent | -3.55 | 3.78 | -0.21 | 0.35 | -7.18 | 3.43 | -0.42 | 0.04 |
| Local self-contained | -4.04 | 3.96 | -0.20 | 0.31 | -7.10 | 3.53 | -0.36 | 0.05 |
|  | Protein g | | | | | | | |
|  | Model 1 (Unadjusted model) | | | | Model 2 (Fully adjusted model) | | | |
|  | B | SE | β | p value | B | SE | β | p value |
| (Constant) | 87.17 | 11.18 |  | 0.00 | 128.22 | 34.80 |  | 0.00 |
| Locally integrated | -16.33 | 11.93 | -0.33 | 0.18 | -15.91 | 12.46 | -0.32 | 0.21 |
| Wider community | -12.40 | 15.81 | -0.12 | 0.44 | -12.41 | 16.31 | -0.12 | 0.45 |
| Family-dependent | -6.93 | 12.44 | -0.12 | 0.58 | -9.74 | 12.76 | -0.17 | 0.45 |
| Local self-contained | -14.49 | 13.02 | -0.22 | 0.27 | -11.65 | 13.14 | -0.18 | 0.38 |
|  | Fats g | | | | | | | |
|  | Model 1 (Unadjusted model) | | | | Model 2 (Fully adjusted model) | | | |
|  | B | SE | β | p value | B | SE | β | p value |
| (Constant) | 72.82 | 16.31 |  | 0.00 | 118.70 | 51.29 |  | 0.02 |
| Locally integrated | -8.38 | 17.41 | -0.12 | 0.63 | -3.62 | 18.37 | -0.05 | 0.84 |
| Wider community | -8.74 | 23.07 | -0.06 | 0.71 | -5.59 | 24.04 | -0.04 | 0.82 |
| Family-dependent | 1.50 | 18.15 | 0.02 | 0.93 | -1.48 | 18.81 | -0.02 | 0.94 |
| Local self-contained | -11.98 | 19.00 | -0.13 | 0.53 | -6.15 | 19.36 | -0.07 | 0.75 |

Adjusted variables: Sex, age, educational status, self -reported health, Index of Multiple Deprivation, number of diseases. B= Unstandardized Beta; SE= Standard error of the mean; β= Standardized Beta*WHO guidance on BMI thresholds for Asian populations (World Health Organization, 2004) was used to categorise BMI of South Asian participants, and the standard BMI categories were used for Caribbean and African participants. Note age is in years*.* **^¥^** This includes all those that are single, separated, divorced and widowed. ^µ^ types of cancer: prostate cancer (70%) and bone cancer (30%); ^∞^Others refers to diseases such kidney diseases, acid reflux, ear and eye problems, and osteoporosis;  ^α^ Significant differences between social networks. **^β ‘^**Others’ referring to mixed ethnicities e.g. African Asians.
