# Supplementary material for: Social networks and their influences on nutrient intake, nutritional status and physical function in community-dwelling ethnically diverse older adults: a mixed-methods longitudinal study
Source: BMC Public Health. 2020 Jun 26;20:1011. doi: 10.1186/s12889-020-09153-y (PMC7318427; doi:10.1186/s12889-020-09153-y)
Supplement: Supplementary file 2 — Additional file 2. Social network changes and nutrient intakes among community-dwelling ethnically diverse older adults (n=81) [file 12889_2020_9153_MOESM2_ESM.docx]

**Additional file 2: Social network changes and nutrient intakes among community-dwelling ethnically diverse older adults (n=81)**

| **Variable*** | **Changed to integrated (n=27)** | **Maintained integrated (n=9)** | **Maintained non-integrated (N=30)** | **Changed to non-integrated (N=15)** | **P value** |
| --- | --- | --- | --- | --- | --- |
| Energy Mean (SD) | 1609.4 (850.6) | 1543.8 (468.0) | 1914.4 (833.3) | 1777.3 (533.5) | 0.241 |
| %TE saturated fat Mean (SD) | 11.2 (3.8) | 9.8 (3.3) | 11.2 (4.3) | 14.0 (4.7) | 0.019 |
| Potassium mg.d^-1^ Mean (SD) | 1959.5 (501.2) | 1709.2 (548.3) | 1501.2 (406.9) | 1412.1 (288.3) | 0.015 |
| Sodium mg.d^-1^ Mean (SD) | 1536.3 (635.6) | 1006.1 (384.6) | 1095.8 (656) | 895.3 (298.7) | 0.030 |
| Folate µg. d^-1^ Mean (SD) | 140.3 (58.4) | 133.3 (45.2) | 115.3 (44.9) | 94.8 (33.6) | 0.032 |

***** showing only nutrients with statistically significant differences (except energy).

TE= Total energy
