# Supplementary material for: Social networks and their influences on nutrient intake, nutritional status and physical function in community-dwelling ethnically diverse older adults: a mixed-methods longitudinal study
Source: BMC Public Health. 2020 Jun 26;20:1011. doi: 10.1186/s12889-020-09153-y (PMC7318427; doi:10.1186/s12889-020-09153-y)
Supplement: Supplementary file 3 — Additional file 3. Association of social networks with MNA-SF, WC, HGS and selected nutrients. [file 12889_2020_9153_MOESM3_ESM.docx]

**Additional file 3: Association of social networks with MNA-SF, WC, HGS and selected nutrients.**

|  | MNA-SF | | | | | | | |
| --- | --- | --- | --- | --- | --- | --- | --- | --- |
|  | Model 1  (Unadjusted model) | | | | Model 2 (Fully adjusted model) | | | |
|  | B | SE | β | p value | B | SE | β | p value |
| (Constant) | 12.20 | 0.76 |  | 0.00 | 16.79 | 2.35 |  | 0.00 |
| Locally integrated | 0.38 | 0.81 | 0.12 | 0.64 | -0.48 | 0.84 | -0.15 | 0.57 |
| Wider community | 0.40 | 1.07 | 0.06 | 0.71 | 0.62 | 1.10 | 0.09 | 0.58 |
| Family-dependent | 0.47 | 0.84 | 0.12 | 0.58 | -0.21 | 0.86 | -0.06 | 0.81 |
| Local self-contained | 0.23 | 0.88 | 0.05 | 0.80 | -0.31 | 0.89 | -0.07 | 0.72 |
|  | Handgrip | | | | | | | |
|  | Model 1 (Unadjusted model) | | | | Model 2 (Fully adjusted model) | | | |
|  | B | SE | β | p value | B | SE | β | p value |
| (Constant) | 25.24 | 4.22 |  | 0.00 | 82.68 | 10.92 |  | 0.00 |
| Locally integrated | 3.63 | 4.51 | 0.19 | 0.42 | -4.06 | 3.91 | -0.21 | 0.30 |
| Wider community | -2.26 | 5.97 | -0.06 | 0.71 | -0.06 | 5.12 | 0.00 | 0.99 |
| Family-dependent | 5.27 | 4.70 | 0.24 | 0.27 | -3.07 | 4.00 | -0.14 | 0.45 |
| Local self-contained | -1.27 | 4.92 | -0.05 | 0.80 | -5.29 | 4.12 | -0.21 | 0.20 |
|  | Waist circumference | | | | | | | |
|  | Model 1 (Unadjusted model) | | | | Model 2 (Fully adjusted model) | | | |
|  | B | SE | β | p value | B | SE | β | p value |
| (Constant) | 108.15 | 4.72 |  | 0.00 | 109.90 | 15.09 |  | 0.00 |
| Locally integrated | -8.26 | 5.04 | -0.39 | 0.11 | -5.19 | 5.40 | -0.25 | 0.34 |
| Wider community | -7.05 | 6.68 | -0.16 | 0.29 | -6.83 | 7.07 | -0.16 | 0.34 |
| Family-dependent | -9.78 | 5.25 | -0.41 | 0.07 | -8.48 | 5.53 | -0.36 | 0.13 |
| Local self-contained | -7.56 | 5.50 | -0.27 | 0.17 | -6.11 | 5.70 | -0.22 | 0.29 |
|  | Energy kcal | | | | | | | |
|  | Model 1 (Unadjusted model) | | | | Model 2 (Fully adjusted model) | | | |
|  | B | SE | β | p value | B | SE | β | p value |
| (Constant) | 1902.60 | 233.72 |  | 0.00 | 2913.00 | 713.97 |  | 0.00 |
| Locally integrated | -173.09 | 249.43 | -0.17 | 0.49 | -213.18 | 255.67 | -0.21 | 0.41 |
| Wider community | -240.30 | 330.53 | -0.11 | 0.47 | -231.26 | 334.58 | -0.11 | 0.49 |
| Family-dependent | -74.70 | 260.06 | -0.06 | 0.78 | -221.52 | 261.80 | -0.19 | 0.40 |
| Local self-contained | -280.81 | 272.28 | -0.21 | 0.31 | -262.92 | 269.56 | -0.19 | 0.33 |
|  | Carbohydrates g | | | | | | | |
|  | Model 1 (Unadjusted model) | | | | Model 2 (Fully adjusted model) | | | |
|  | B | SE | β | p value | B | SE | β | p value |
| (Constant) | 229.65 | 28.64 |  | 0.00 | 319.08 | 88.19 |  | 0.00 |
| Locally integrated | -7.24 | 30.56 | -0.06 | 0.81 | -22.67 | 31.58 | -0.18 | 0.48 |
| Wider community | -24.11 | 40.50 | -0.09 | 0.55 | -28.28 | 41.33 | -0.11 | 0.50 |
| Family-dependent | -7.20 | 31.86 | -0.05 | 0.82 | -30.82 | 32.34 | -0.21 | 0.34 |
| Local self-contained | -36.03 | 33.36 | -0.22 | 0.28 | -44.34 | 33.30 | -0.27 | 0.19 |
|  | Fibre g | | | | | | | |
|  | Model 1 (Unadjusted model) | | | | Model 2 (Fully adjusted model) | | | |
|  | B | SE | β | p value | B | SE | β | p value |
| (Constant) | 20.91 | 3.40 |  | 0.00 | 20.23 | 9.36 |  | 0.03 |
| Locally integrated | -3.39 | 3.62 | -0.23 | 0.35 | -6.49 | 3.35 | -0.43 | 0.06 |
| Wider community | 0.77 | 4.80 | 0.03 | 0.87 | -1.87 | 4.39 | -0.06 | 0.67 |
| Family-dependent | -3.55 | 3.78 | -0.21 | 0.35 | -7.18 | 3.43 | -0.42 | 0.04 |
| Local self-contained | -4.04 | 3.96 | -0.20 | 0.31 | -7.10 | 3.53 | -0.36 | 0.05 |
|  | Protein g | | | | | | | |
|  | Model 1 (Unadjusted model) | | | | Model 2 (Fully adjusted model) | | | |
|  | B | SE | β | p value | B | SE | β | p value |
| (Constant) | 87.17 | 11.18 |  | 0.00 | 128.22 | 34.80 |  | 0.00 |
| Locally integrated | -16.33 | 11.93 | -0.33 | 0.18 | -15.91 | 12.46 | -0.32 | 0.21 |
| Wider community | -12.40 | 15.81 | -0.12 | 0.44 | -12.41 | 16.31 | -0.12 | 0.45 |
| Family-dependent | -6.93 | 12.44 | -0.12 | 0.58 | -9.74 | 12.76 | -0.17 | 0.45 |
| Local self-contained | -14.49 | 13.02 | -0.22 | 0.27 | -11.65 | 13.14 | -0.18 | 0.38 |
|  | Fats g | | | | | | | |
|  | Model 1 (Unadjusted model) | | | | Model 2 (Fully adjusted model) | | | |
|  | B | SE | β | p value | B | SE | β | p value |
| (Constant) | 72.82 | 16.31 |  | 0.00 | 118.70 | 51.29 |  | 0.02 |
| Locally integrated | -8.38 | 17.41 | -0.12 | 0.63 | -3.62 | 18.37 | -0.05 | 0.84 |
| Wider community | -8.74 | 23.07 | -0.06 | 0.71 | -5.59 | 24.04 | -0.04 | 0.82 |
| Family-dependent | 1.50 | 18.15 | 0.02 | 0.93 | -1.48 | 18.81 | -0.02 | 0.94 |
| Local self-contained | -11.98 | 19.00 | -0.13 | 0.53 | -6.15 | 19.36 | -0.07 | 0.75 |

Adjusted variables: Sex, age, educational status, self -reported health, Index of Multiple Deprivation, number of diseases. B= Unstandardized Beta; SE= Standard error of the mean; β= Standardized Beta*WHO guidance on BMI thresholds for Asian populations (World Health Organization, 2004) was used to categorise BMI of South Asian participants, and the standard BMI categories were used for Caribbean and African participants. Note age is in years*.* **^¥^** This includes all those that are single, separated, divorced and widowed. ^µ^ types of cancer: prostate cancer (70%) and bone cancer (30%); ^∞^Others refers to diseases such kidney diseases, acid reflux, ear and eye problems, and osteoporosis;  ^α^ Significant differences between social networks. **^β ‘^**Others’ referring to mixed ethnicities e.g. African Asians.
